# Genetically predicted cortisol levels and risk of venous thromboembolism

**DOI:** 10.1371/journal.pone.0272807

**Published:** 2022-08-19

**Authors:** Elias Allara, Wei-Hsuan Lee, Stephen Burgess, the INVENT consortium, Susanna C. Larsson

**Affiliations:** 1 BHF Cardiovascular Epidemiology Unit, Department of Public Health and Primary Care, University of Cambridge, Cambridge, United Kingdom; 2 MRC Biostatistics Unit, University of Cambridge, Cambridge, United Kingdom; 3 Unit of Medical Epidemiology, Department of Surgical Sciences, Uppsala University, Uppsala, Sweden; 4 Unit of Cardiovascular and Nutritional Epidemiology, Institute of Environmental Medicine, Karolinska Institutet, Stockholm, Sweden; Albert Einstein College of Medicine, UNITED STATES

## Abstract

**Introduction:**

In observational studies, venous thromboembolism (VTE) has been associated with Cushing’s syndrome and with persistent mental stress, two conditions associated with higher cortisol levels. However, it remains unknown whether high cortisol levels within the usual range are causally associated with VTE risk. We aimed to assess the association between plasma cortisol levels and VTE risk using Mendelian randomization.

**Methods:**

Three genetic variants in the *SERPINA1*/*SERPINA6* locus (rs12589136, rs11621961 and rs2749527) were used to proxy plasma cortisol. The associations of the cortisol-associated genetic variants with VTE were acquired from the INVENT (28 907 cases and 157 243 non-cases) and FinnGen (6913 cases and 169 986 non-cases) consortia. Corresponding data for VTE subtypes were available from the FinnGen consortium and UK Biobank. Two-sample Mendelian randomization analyses (inverse-variance weighted method) were performed.

**Results:**

Genetic predisposition to higher plasma cortisol levels was associated with a reduced risk of VTE (odds ratio [OR] per one standard deviation increment 0.73, 95% confidence interval [CI] 0.62–0.87, p<0.001). The association was stronger for deep vein thrombosis (OR 0.69, 95% CI 0.55–0.88, p = 0.003) than for pulmonary embolism which did not achieve statistical significance (OR 0.83, 95% CI 0.63–1.09, p = 0.184). Adjusting for genetically predicted systolic blood pressure inverted the direction of the point estimate for VTE, although the resulting CI was wide (OR 1.06, 95% CI 0.70–1.61, p = 0.780).

**Conclusions:**

This study provides evidence that genetically predicted plasma cortisol levels in the high end of the normal range are associated with a decreased risk of VTE and that this association may be mediated by blood pressure. This study has implications for the planning of observational studies of cortisol and VTE, suggesting that blood pressure traits should be measured and accounted for.

## Introduction

Cortisol is a glucocorticoid hormone that regulates numerous physiologic processes in the body, such as the stress and immune responses, intermediary metabolism, cardiovascular function, skeletal growth, reproduction, and cognition [1]. Abnormally high cortisol levels are a defining feature of Cushing’s syndrome (CS), a medical condition which manifests clinically with a range of conditions such as abdominal adiposity and hypertension [2–4].

The relationship between cortisol and venous thromboembolism (VTE) is not fully understood. VTE occurs more frequently in patients with CS than in the general population [5, 6], and mechanistic studies show evidence of hypercoagulable state in CS patients compared to healthy controls [6]. A cohort study found a positive association between VTE and persistent mental stress in the previous 1 to 5 years in a sample drawn from the general population [7]. Because chronic psychological stress is associated with higher cortisol concentration, this study provides indirect evidence relating to a potential association between chronic hypercortisolemia and VTE risk in the general population. Additionally, a meta-analysis of observational studies shows that normal-range greater cortisol levels are associated with greater risk of cardiovascular disease (CVD), and this finding is substantially confirmed by genetic evidence [8]. Because CVD and VTE share several risk factors, an association between usual-range cortisol and VTE seems possible.

We conducted a Mendelian randomization (MR) investigation of the association between circulating cortisol levels and risk of VTE in the general population and assessed whether the association was mediated by abdominal adiposity and blood pressure. In MR studies, genetic variants reliably related to the risk factor are used as instrumental variables with the purpose to evaluate the causal relationship between a risk factor and a disease. Confounding and reverse causality are diminished in MR studies because the genetic alleles are randomly allocated at conception and cannot be altered by the development of disease. Understanding whether cortisol is causally associated with VTE risk may offer initial evidence as to whether sustained elevated cortisol levels within the usual range affect the risk of VTE. This study is an extension of our previous MR study showing a positive association of plasma cortisol levels proxied by three genetic variants with atrial fibrillation risk, an association that was mediated by abdominal adiposity and systolic blood pressure [9].

## Materials and methods

### Study design

**Fig 1:**
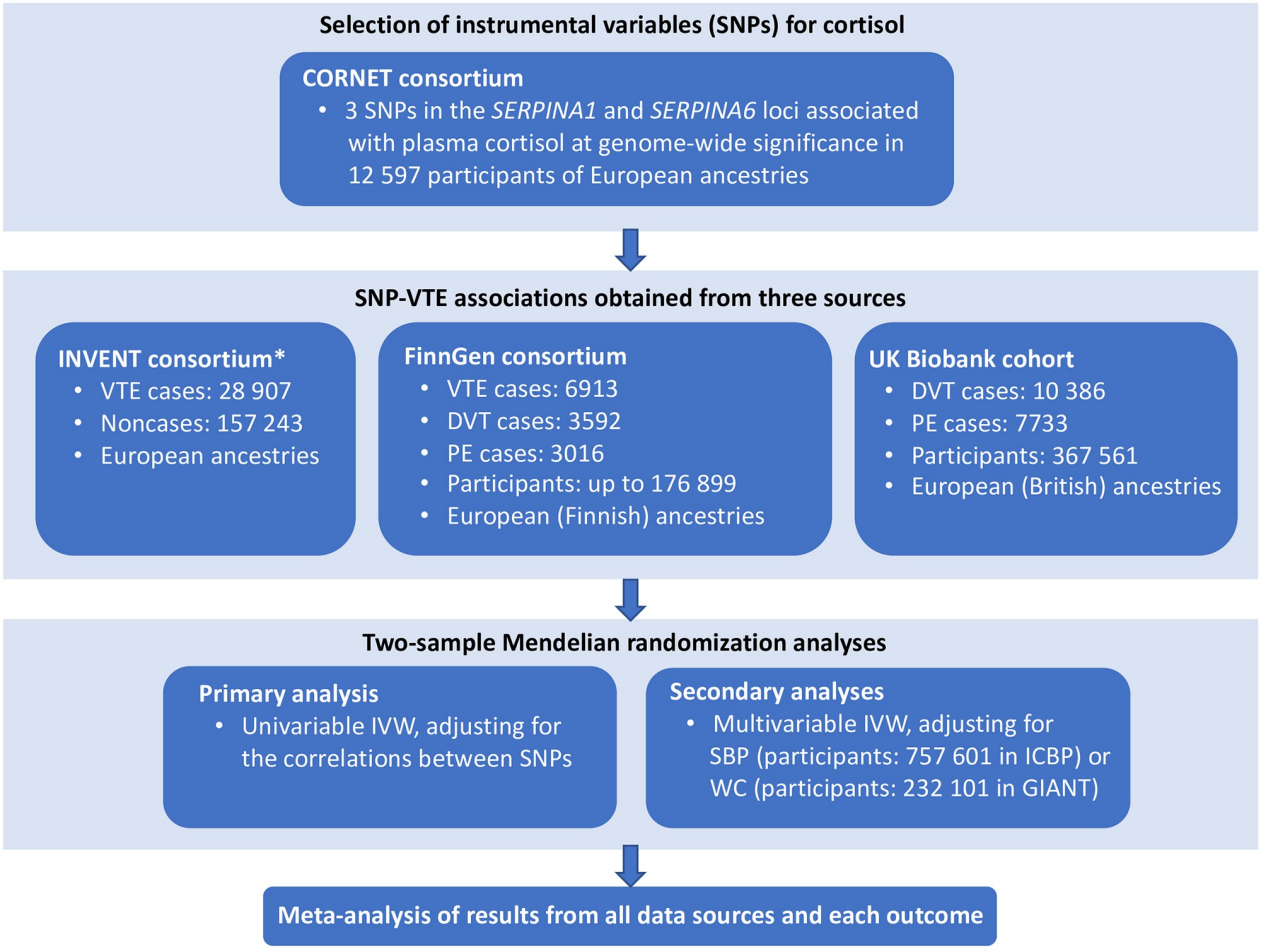
Study overview. Abbreviations: CORNET, CORtisol NETwork; INVENT, International Network Against Venous Thrombosis; DVT, deep vein thrombosis; GIANT, Genetic Investigation of ANthropometric Traits consortium; ICBP, International Consortium of Blood Pressure; IVW, inverse-variance weighted; PE, pulmonary embolism; SBP, systolic blood pressure; SNP, single-nucleotide polymorphisms; VTE, venous thromboembolism; WC, waist circumference. *The INVENT consortium includes participants from the UK Biobank study.

We used a two-sample Mendelian randomization study design, where summary statistics data for the genetic associations with plasma cortisol were acquired from the CORtisol NETwork (CORNET) consortium and genetic associations with VTE overall or its subtypes were obtained from the International Network Against Venous Thrombosis (INVENT) consortium (recruited between 1972–2014), FinnGen consortium (2017-ongoing) and the UK Biobank cohort (2006–2010). The study overview is depicted in Fig 1. Ethics approval is not required for this study as it relies on summary associations from published studies. Information on participant characteristics and ethics approval for the individual studies included in this analysis is available in the respective publications of the CORNET [10], INVENT [11] and FinnGen [12] studies.

We have reported summarized demographic data for individual cohorts participating to CORNET and INVENT, for which this information was available (S1 Table).

### Genetic instruments for cortisol

The instrument for cortisol comprised three genetic variants (single nucleotide polymorphisms; SNPs) in the region of chromosome 14, containing the *SERPINA6* and *SERPINA1* genes, that were related to morning plasma cortisol levels at *P*<5×10^−8^ in the CORNET consortium (rs12589136, rs11621961 and rs2749527) [10]. The discovery genome-wide association meta-analysis included nine studies with a total of 12 597 participants of European descents [10]. Mean morning plasma cortisol levels in the included studies ranged from 305 to 765 nmol/L. The genetic association estimates were adjusted for age, sex, and genetic principal components, when available in the individual studies [10]. The genes *SERPINA6* and *SERPINA1* are expressed in tissues (e.g. the pancreas and liver) that have a known role in cortisol physiology [13], confirming the reliability of the genetic variants as predictors of cortisol levels.

### Data sources for VTE and mediators

Summary statistics of the three above-mentioned SNPs with VTE in individuals of European ancestries were acquired from the INVENT (which includes the UK Biobank study) [11] and FinnGen [12] studies (Fig 1). The corresponding data for VTE subtypes were acquired from the FinnGen consortium and the UK Biobank, and the equivalent data for waist circumference (as a measure of abdominal adiposity) and systolic blood pressure were taken from the Genetic Investigation of ANthropometric Traits consortium [14] and the International Consortium of Blood Pressure [15], respectively (Fig 1). The INVENT consortium included data from 18 prospective cohorts and case-control studies of adult women and men of European ancestry or African American ancestry [11]. In the present analysis of cortisol and VTE, we only included data from the analysis of individuals of European ancestries to avoid population stratification bias. In the INVENT consortium, the genetic association estimates were adjusted for study-specific covariates and participant relatedness, when available in the individual studies [11]. The FinnGen consortium (R4 release) included 176 899 participants of Finnish descent. Participants with high genotype missingness (>5%), extreme heterozygosity (±4 standard deviation), uncertain gender or of non-Finnish origin were excluded [12]. The genetic association estimates were adjusted for age, sex, ten genetic principal components, and genotyping batch [12]. Relevant participant consent and ethical approval were previously obtained in all the studies analyzed.

### Power calculation

Power calculation were performed using an online web resource [16], assuming 0.05 significance level and 0.54% variance explained by the genetic instrument [10]. Owing to the absence of prior studies measuring the relation between cortisol and VTE, we anticipated an odds ratio (OR) of ~1.4, based on the in multivariable analyses of an observational investigation into stress and VTE [7]. Results of power calculations show power greater than 80% in all pooled analyses for VTE and both subtypes (Table 1).

**Table 1:** Power calculation.

Outcome	Data source	Cases	Controls	Total	Power
Venous thromboembolism	INVENT	28 907	157 243	186 150	99.2%
	FinnGen	6 913	169 986	176 899	65.8%
	Meta-analysis	35 820	327 229	363 049	99.9%
Deep vein thrombosis	FinnGen	3 592	173 307	176 899	41.0%
	UK Biobank	10 386	357 175	367 561	83.3%
	Meta-analysis	13 978	530 482	544 460	92.5%
Pulmonary embolism	FinnGen	3 016	173 883	176 899	35.7%
	UK Biobank	7 733	359 828	367 561	71.9%
	Meta-analysis	10 749	533 711	544 460	85.0%

### Statistical analysis

We conducted two-sample MR analyses in R using the MendelianRandomization package v. 0.5.6 [17]. There was no overlap between exposure and outcomes samples. The fixed-effect inverse-variance weighted approach was applied to all analyses, and adjustment was made for linkage disequilibrium (LD) between genetic variants. The LD matrix was estimated in 367 643 individuals of European descents in the UK Biobank study using qctool v2.0.5. To evaluate whether waist circumference and blood pressure may mediate the potential association between genetically predicted plasma cortisol and VTE, we conducted multivariable fixed-effect MR analyses. Results across data sources were combined through meta-analysis after MR analysis.

## Results

Characteristics of the three genetic variants (rs12589136, rs11621961 and rs2749527) applied as instrumental variables for plasma cortisol levels and their relations with VTE in the INVENT and FinnGen consortia are presented in Table 2.

**Table 2:** Characteristics of the genetic variants used as instrumental variables for cortisol levels and their associations with venous thromboembolism in the INVENT and FinnGen consortia.

rsID	Chr	Gene	EA	Plasma cortisol levels			INVENT consortium			FinnGen consortium		
				Beta (SE)*	*P*	EAF	Beta (SE)*	*P*	EAF	Beta (SE)*	*P*	EAF
rs12589136	14	*SERPINA6*	T	0.22	0.10 (0.015)	3.3×10^−12^	0.21	-0.01 (0.01)	0.24	0.21	-0.05 (0.02)	0.03
rs11621961	14	*SERPINA6*	C	0.64	0.08 (0.013)	4.0×10^−8^	0.63	-0.04 (0.01)	4.0×10^−5^	0.65	-0.01 (0.02)	0.44
rs2749527	14	*SERPINA1*	C	0.51	0.08 (0.013)	5.2×10^−11^	0.49	-0.02 (0.01)	0.02	0.49	-0.01 (0.02)	0.55

Abbreviations: Chr, chromosome; EA, effect allele; EAF, effect allele frequency; INVENT, International Network Against Venous Thrombosis; SE, standard error.

*The beta coefficients and standard errors correspond to the age- and sex-adjusted cortisol z-score change in cortisol levels for each additional effect allele.

**Fig 2:**
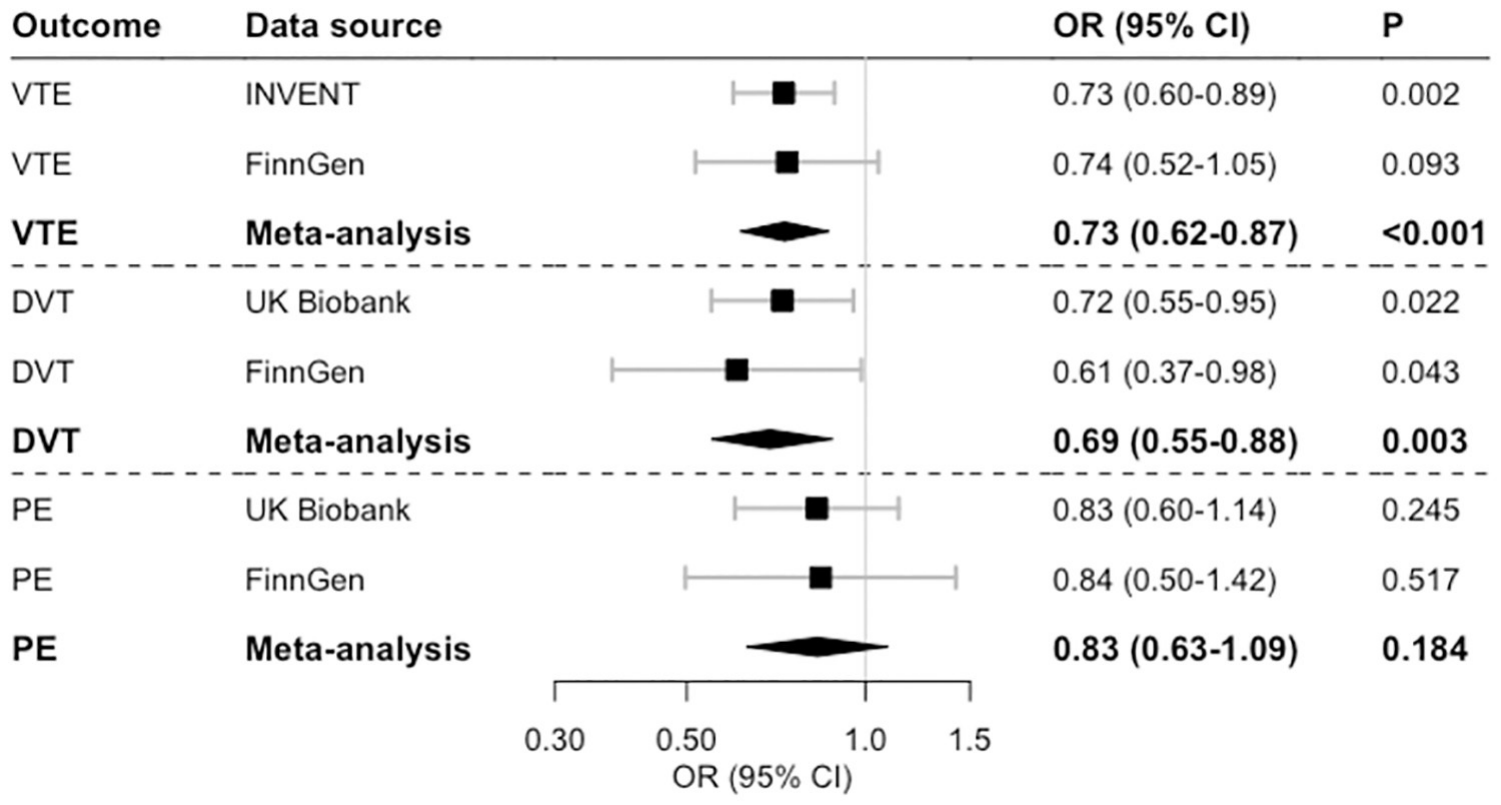
Associations of genetically predicted one standard deviation increment of plasma cortisol levels with risk of venous thromboembolism (VTE), deep vein thrombosis (DVT), and pulmonary embolism (PE). Abbreviations: CI, confidence interval; INVENT, International Network Against Venous Thrombosis; OR, odds ratio.

Higher genetically predicted cortisol levels were related to a decreased risk of VTE. The OR of VTE in the meta-analysis of results from the INVENT and FinnGen consortia was 0.73 (95% confidence intervals [CI] 0.62–0.87, p<0.001) (Fig 2). Associations were similar in both data sources. The association was stronger for deep vein thrombosis (OR 0.69, 95% CI 0.55–0.88, p = 0.003) than for pulmonary embolism which did not achieve statistical significance (OR 0.83, 95% CI 0.63–1.09, p = 0.184).

**Fig 3:**
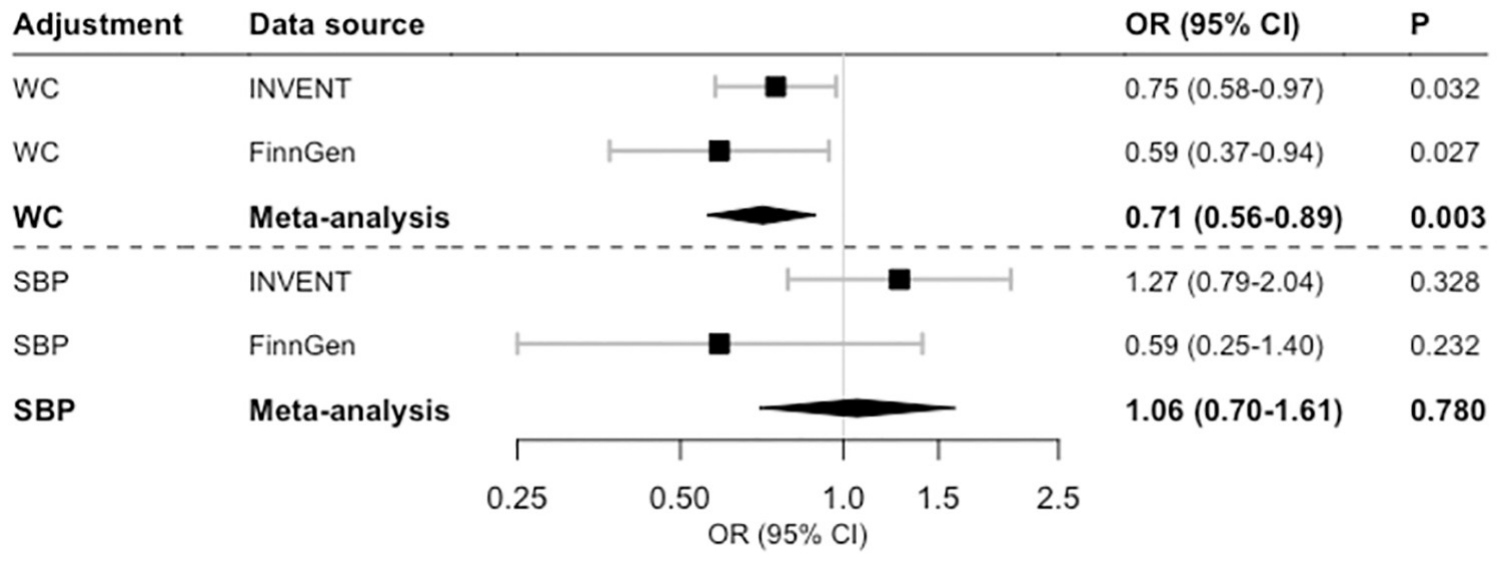
Association of genetically predicted one standard deviation increment of plasma cortisol levels with risk of venous thromboembolism in multivariable Mendelian randomization analysis adjusted for genetically predicted waist circumference (WC) or systolic blood pressure (SBP). Abbreviations: CI, confidence interval; INVENT, International Network Against Venous Thrombosis; OR, odds ratio.

The relation between genetically predicted cortisol levels and VTE risk was not appreciably altered after adjustment for genetically predicted waist circumference (OR 0.71, 95% CI 0.56–0.89, p = 0.003) but did not remain after adjustment for genetically predicted systolic blood pressure (OR 1.06, 95% CI 0.70–1.61, p = 0.780) (Fig 3). Individual-variant genetic associations with systolic blood pressure are available in S2 Table.

## Discussion

Findings of this first MR investigation of the possible causal relationship between cortisol and VTE showed that genetic predisposition to high plasma cortisol levels is related to a decreased risk of VTE, particularly deep vein thrombosis. Accounting for genetically predicted systolic blood pressure inverted the direction of the point estimate, in keeping with previous observational evidence, although the adjusted association remains inconclusive in this study. While the relation between blood pressure and VTE is not fully elucidated, higher systolic blood pressure was found to be associated with a lower risk of VTE in the UK Biobank study [18, 19] and in 5.5 million primary care patients [20]. It is therefore possible that blood pressure may mediate the association between genetically predicted cortisol and risk of VTE as this association did not persist upon adjustment for systolic blood pressure in the multivariable MR analysis. The apparent inconsistency between the current study and published observational studies might be explained by treatment with antihypertensive medications in a sizeable proportion of participants. Medical treatment of hypertension is a priority in patients with CS [21], and the inverse association between cortisol and VTE driven by raised blood pressure may be offset by antihypertensive medications, resulting in an apparent positive association of hypercortisolemia with VTE risk. However, as the genetic associations with VTE were estimated in datasets taken from the general population, the results of our investigation do not primarily relate to individuals with CS, but rather to those with cortisol levels in the usual range.

Our findings for cortisol and VTE are in opposite direction to our recent results for cortisol, proxied by the same genetic variants as in the present study, and risk of atrial fibrillation [9]. In contrast to VTE, systolic blood pressure is positively associated with risk of atrial fibrillation [18, 22]. In our previous MR study, the association between genetic predisposition to higher cortisol levels and increased risk of atrial fibrillation was partly mediated by systolic blood pressure and partly by abdominal adiposity [9].

A strength of this study is that results of MR analyses are less likely to be biased by reverse causality and residual confounding compared with traditional observational studies. In the observational setting, particularly in a case-control study, it may not be possible to decipher whether elevated cortisol levels increase the risk of VTE or whether VTE has caused increased internal stress and consequential elevation of circulating cortisol. This reverse causation bias is avoided in MR studies as the genetic instruments are set at conception and cannot be altered by the disease. Furthermore, confounding from factors that can modify cortisol levels was reduced in this MR study as genetic alleles are randomly distributed at conception. Hence, one trait (e.g., plasma cortisol) is unlikely associated with other traits (e.g., confounders). A further strength is the use of data from large-scale genetic consortia and biobanks, thereby providing large number of VTE cases and high precision of the estimates. Our results were consistent across data sources which strengthen the evidence that the observed association between plasma cortisol and VTE is causal.

A constraint of this MR study is that the genetic variants used as instrumental variables accounted for only 0.54% of the variation in morning plasma cortisol. This low variation resulted in decreased statistical power in our analyses. However, given the large sample sizes of the outcome datasets, we had high precision in our MR estimates as reflected by relatively narrow CIs. Another shortcoming is that we cannot entirely rule out that the genetic variants used as instrumental variables for cortisol affect the risk of VTE directly through any other unknown causal pathway. As all the genetic variants are in the same gene region, it is possible that the genetic associations with VTE risk are driven by another pathway regulated at this locus. In this regard, we note that the cortisol-raising genetic variants used in our study were inversely associated at a genome-wide level of significance with albumin and sex hormone-binding globulin. However, these biomarkers have not been shown to be positively associated with VTE risk in observational epidemiological analyses [23, 24], rendering them unlikely to affect the inverse association between cortisol and VTE risk observed in this study. A potential limitation is that our analyses only included participants of European ancestries which limits the generalizability of our results to populations of non-European origin. A further potential limitation of our study concerns the moderate LD (>0.2) observed for two of the three instrumental variables (rs12689136 and rs2749527). We have however addressed this at the analysis stage, by explicitly accounting for between-variant LD in all MR analyses. Finally, a limitation of this work is that covariate adjustment in the INVENT consortium was somewhat inconsistent; out of the 16 studies, 3 did not adjust for age and sex, and 1 additionally adjusted for hypertension [11]. However, results were consistent in FinnGen, which adjusted for age and sex and did not adjust for hypertension, suggesting that these variations in covariate adjustment are unlikely to affect the overall conclusion of this study.

In conclusion, the present MR study provides evidence that genetically predicted plasma cortisol levels in the high end of the usual range are associated with a reduced risk of VTE and that this association may be mediated by blood pressure. This finding needs confirmation in further MR and longitudinal prospective studies. While this information *per se* is unlikely to have immediate clinical implications, it has implication for research, as it prompts the planning of observational studies of cortisol and VTE that measure and account for blood pressure traits.

## Supporting information

S1 Table Summary demographic characteristics of the CORNET and INVENT consortia. SD, standard deviation.
(DOCX)

S2 Table Individual-variant genetic associations with systolic blood pressure (ICBP study) used in the present study’s multivariable Mendelian randomization analysis. (DOCX)
