# Supplementary material for: Genetically predicted cortisol levels and risk of venous thromboembolism
Source: PLoS One. 2022 Aug 19;17(8):e0272807. doi: 10.1371/journal.pone.0272807 (PMC9390895; doi:10.1371/journal.pone.0272807)
Supplement: S1 Table — SD, standard deviation. (DOCX) [file pone.0272807.s001.docx]

|  | **Full study name** | **Acronym** | **Sample size** | | **Female (%)** | | **Age in years** | | | **Plasma cortisol in nmol/l** | | |  |
| --- | --- | --- | --- | --- | --- | --- | --- | --- | --- | --- | --- | --- | --- |
|  |  |  | **Cases** | **Total** | |  | | **Mean (SD)** | **Range** | | **Mean (SD)** | **Range** | |
| **CORNET** | Orkney Complex Disease Study | ORCADES | - | 886 | | 55 | | 53.5 (15.7) | 17-97 | | 765 (315) | 11-3641 | |
|  | 100001 Dalmations The Croatian Biobank | CROATIA-Korcula | - | 898 | | 64 | | 56.2 (13.9) | 18-98 | | 698 (207) | 59-815 | |
|  |  | CROATIA-Split | - | 496 | | 57 | | 45.0 (14.7) | 18-85 | | 979 (404) | 94-2831 | |
|  |  | CROATIA-Vis | - | 892 | | 57 | | 56.4 (15.5) | 18-93 | | 622 (230) | 64-1820 | |
|  | Rotterdam Study | Rotterdam Study | - | 2,945 | | 56 | | 71.9 (7.0) | 61-105 | | 305 (94) | 5-679 | |
|  | Helsinki Birth Cohort 1934-44 Study | HBCS1934-44 | - | 451 | | 64 | | 60.6 (2.8) | 56 - 67 | | 393 (120) | 125-990 | |
|  | Northern Finland Birth Cohort 1966 | NFBC1966 | - | 1,192 | | 100 | | 31 (0) | n/a | | 380 (160) | 40-2370 | |
|  | Avon Longitudinal Study of Parents and Children | ALSPAC | - | 1,567 | | 50 | | 15.4 (0.3) | 14-17 | | 486 (174) | 58-1683 | |
|  | Invecchiare in Chianti, aging in Chianti | InChianti | - | 1,210 | | 56 | | 68.3 (15.6) | 21-102 | | 375 (135) | 19-1291 | |
|  | Prospective Investigation of the Vasculature in Uppsala Seniors | PIVUS | - | 919 | | 50 | | 70.2 (0.17) | 69-72 | | 386 (125) | 31-930 | |
|  | Prevention of Renal and Vascular End-stage Disease | PREVEND | - | 1,151 | | 49 | | 49.4 (13.0) | 28-75 | | 442 (201) | 20-1734 | |
| **INVENT (European-ancestry participants)** | Atherosclerosis Risk in Communities study | ARIC | 241 | 8,887 | | 55 | | 54.0 (5.7) | 45-64 | | - | - | |
|  | Cardiovascular Health Study | CHS | 95 | 3,119 | | 60.3 | | 72.3 (5.4) | 64-98 | | - | - | |
|  | Early-Onset Venous Thrombosis | EOVT | 411 | 1,639 | | 44 (cases) ; 70 (controls) | | 36.0 (9.0) (cases); 50.0 (6.0) (controls) | - | | - | - | |
|  | eMERGE | eMERGE | 1,558 | 11,585 | | 53 | | - | - | | - | - | |
|  | Framingham Heart Study | FHS | 222 | 7,851 | | 55 | | 52.6 (16.4) | 19-100 | | - | - | |
|  | Heart and Vascular Health | HVH | 1,684 | 3,325 | | 67 (cases); 65 (controls) | | 63.1 (14.2) (cases); 65.5 (11.4) (controls) | 18-89 | | - | - | |
|  | HUNT Study | HUNT | 811 | 5,203 | | 48 | | 68.6 (14.7) | 21-101 | | - | - | |
|  | JUPITER | JUPITER | 77 | 8,749 | | 32 | | 66.1 (7.8) | 50-93 | | - | - | |
|  | MARseille THrombosis Association study | MARTHA | 1,542 | 2,652 | | 66 (cases) ; 69 (controls) | | 40.9 (15.7) (cases); 68.1 (2.2) (controls) | 1-90 (cases); 65-83 (controls) | | - | - | |
|  | Mayo Study | MAYO | 1,238 | 2,525 | | 51 | | 55.0 (16.0) | 18-95 | | - | - | |
|  | Multiple Environmental and Genetic Assessment of risk factors for venous thrombosis | MEGA | 1,289 | 2,338 | | 51 (cases); 56 (controls) | | 48.2 (12.8) (cases); 76.2 (5.4) ((controls) | 18-69 (cases); 65-98 (controls) | | - | - | |
|  | Nurses Health Study, Nurses Health Study II and Health Professional Follow-Up Study | NHS/NHSII/HPFS | 4,636 | 37,664 | | 64 | | 58.3 (9.9) | 32-95 | | - | - | |
|  | Tromsø Study | Tromsø Study | 528 | 1,054 | | 52 | | 58.1 (13.7) | 25-90 | | - | - | |
|  | UK Biobank | UK Biobank | 13,863 | 58,202 | | 54 | | 59.6 (7.1) | 40-71 | | - | - | |
|  | Women's Genome Health Study | WGHS | 618 | 22,650 | | 100 | | 54.2 (7.1) | 45-89 | | - | - | |
|  | Women's Health Initiative | WHI | 622 | 9,761 | | 100 | | 67.0 (6.4) | 50-79 | | - | - | |
