# Supplementary material for: Genetically predicted cortisol levels and risk of venous thromboembolism
Source: PLoS One. 2022 Aug 19;17(8):e0272807. doi: 10.1371/journal.pone.0272807 (PMC9390895; doi:10.1371/journal.pone.0272807)
Supplement: S2 Table — (DOCX) [file pone.0272807.s002.docx]

|  |  |  |  | **Systolic blood pressure** | | |
| --- | --- | --- | --- | --- | --- | --- |
| **rsID** | **Chr** | **Gene** | **EA** | **EAF** | **Beta (SE)*** | ***P*** |
| rs12589136 | 14 | *SERPINA6* | T | 0.21 | 0.05 (0.037) | 0.22 |
| rs11621961 | 14 | *SERPINA6* | C | 0.63 | 0.07 (0.031) | 0.04 |
| rs2749527 | 14 | *SERPINA1* | C | 0.49 | 0.04 (0.030) | 0.16 |
